# Developing a Preoperative Algorithm for the Diagnosis of Uterine Leiomyosarcoma

**DOI:** 10.3390/diagnostics10100735

**Published:** 2020-09-23

**Authors:** Hannah Lawlor, Alexandra Ward, Alison Maclean, Steven Lane, Meera Adishesh, Sian Taylor, Shandya Bridget DeCruze, Dharani Kosala Hapangama

**Affiliations:** 1 Department of Women’s and Children’s Health, Institute of Life Course and Medical Sciences, University of Liverpool Member of Liverpool Health Partners, Liverpool L8 7SS, UK; hll15@hotmail.co.uk (H.L.); Alexandra.ward294@gmail.com (A.W.); alison.maclean@liverpool.ac.uk (A.M.); M.Adishesh@liverpool.ac.uk (M.A.); 2 Department of Biostatistics, University of Liverpool Member of Liverpool Health Partners, Liverpool L69 3BX, UK; slane@liverpool.ac.uk; 3 Liverpool Women’s NHS Foundation Trust Member of Liverpool Health Partners, Liverpool L8 7SS, UK; sian.taylor@lwh.nhs.uk (S.T.); shandyabdecruze@gmail.com (S.B.D.)

**Keywords:** fibroid, leiomyoma, uterine leiomyosarcoma, uterine neoplasm, diagnosis

## Abstract

Early diagnosis of the rare and life-threatening uterine leiomyosarcoma (LMS) is essential for prompt treatment, to improve survival. Preoperative distinction of LMS from benign leiomyoma remains a challenge, and thus LMS is often diagnosed post-operatively. This retrospective observational study evaluated the predictive diagnostic utility of 32 preoperative variables in 190 women who underwent a hysterectomy, with a postoperative diagnosis of leiomyoma (*n* = 159) or LMS (*n* = 31), at the Liverpool Women’s National Health Service (NHS) Foundation Trust, between 2010 and 2019. A total of 7 preoperative variables were associated with increased odds of LMS, including postmenopausal status (*p* < 0.001, OR 3.08), symptoms of pressure (*p* = 0.002, OR 2.7), postmenopausal bleeding (*p* = 0.001, OR 5.01), neutrophil count ≥7.5 × 10^9^/L (*p* < 0.001, OR 5.72), haemoglobin level <118 g/L (*p* = 0.037, OR 2.22), endometrial biopsy results of cellular atypia or neoplasia (*p* = 0.001, OR 9.6), and a mass size of ≥10 cm on radiological imaging (*p* < 0.0001, OR 8.52). This study has identified readily available and easily identifiable preoperative clinical variables that can be implemented into clinical practice to discern those with high risk of LMS, for further specialist investigations in women presenting with symptoms of leiomyoma.

## 1. Introduction

Uterine leiomyomas, more commonly known as fibroids, are benign smooth muscle tumours of the uterus, which may cause pressure, heavy menstrual bleeding, pelvic pain, or be asymptomatic [1]. Usually identified in women of reproductive age, leiomyomas are common, with a reported prevalence ranging from 25 to 77% [2], and their incidence increases with advancing age [3]. A rare uterine malignancy, uterine leiomyosarcoma (LMS) shares many common features with benign uterine leiomyomas. LMS accounts for approximately 1–2% of uterine malignancies with a prevalence of 0.64 per 100,000 women [4]. However, the incidence of LMS in women undergoing a hysterectomy for suspected leiomyoma is much higher, at 1 per 1000 [5]. Although LMS occurs more frequently in postmenopausal women, in premenopausal women, key LMS-associated symptoms include vaginal bleeding, pelvic pain and pressure, thus clinical symptomatology is shared with leiomyomas [1,3,4]. Preoperative diagnosis of LMS is challenging, and currently there are no reliable diagnostic tools that are adequately sensitive enough to differentiate between LMS and benign uterine leiomyomas [6]. Most affected women are thus diagnosed postoperatively after histological examination of the uterus [7], which results in delayed and/or inappropriate treatment. Uterine leiomyomas are frequently managed conservatively, with a focus on symptom control, or with minimally invasive, limited surgical/nonsurgical procedures to preserve fertility, such as uterine artery embolization, magnetic resonance image (MRI)-guided transcutaneous focused ultrasound [8] and myomectomy. However, all of the above conservative treatments permit disease progression in a misdiagnosed LMS and can contribute to further reduction in cancer survival (12–25% 5-year survival rate) [5,9,10]. Uterine leiomyoma is the indication in approximately 30–42% of all hysterectomies conducted for benign conditions [11], and laparoscopic route is preferred to decrease associated surgical morbidity and recovery time [12]. However, a laparoscopic approach may necessitate morcellation of the larger fibroid uteri [13]. If performed on an undiagnosed LMS, this poses the risk of dissemination, leading to iatrogenic upstaging of malignancy [14], and lower disease-free survival and poorer overall survival rate (10.8 months compared to 39.6 months) [15].

Accurate preoperative diagnosis of uterine LMS is needed to improve outcomes for women with LMS, and there is limited research in this area. Transvaginal ultrasound (TVUS) is frequently the first imaging modality employed to investigate uterine pathology; however, the sensitivity for detecting LMS is low, mostly due to similarities in sonographic appearance with leiomyomas [16,17]. Certain imaging features on computed tomography (CT) or magnetic resonance imaging (MRI) that are considered to be suggestive of malignancy have been investigated [18,19,20]; however, imaging characteristics that are highly predictive of LMS remain largely undefined. Elevated levels of tumour biomarkers cancer antigen 125 (CA-125) and lactate dehydrogenase (LDH) can be associated with malignancy, and they are often used in investigation of LMS despite poor sensitivity and specificity [21,22]. There are limited published data on the use of endometrial biopsy in diagnosis of LMS; however, some studies report diagnostic accuracy of up to 64% in LMS [23]. There is some evidence for the use of these multiple investigations in conjunction with each other to increase the predictive diagnostic value for LMS [24].

The main aim of this study was to evaluate the clinical predictive value of routinely and universally available preoperative variables to differentiate between malignant uterine LMS and benign leiomyomas in a cohort of women undergoing hysterectomy for symptomatic leiomyoma, and we subsequently identified 7 preoperative variables which can be used to stratify their risk of a diagnosis of malignant LMS, to consider further more advanced, specialist investigations such as MRI.

## 2. Materials and Methods

We conducted a retrospective observational study of two groups of women who had undergone a hysterectomy for symptomatic leiomyoma. Group 1 included all consecutive women who had been postoperatively diagnosed with LMS on histology, who underwent surgery between 2010 and 2019 (*n* = 31), and group 2 consisted of all consecutive women who underwent hysterectomy due to symptomatic leiomyoma, confirmed on histology, over a 12 month period from January to December in 2016 (*n* = 159). This was a single-site study, based at the Liverpool Women’s NHS Foundation Trust (LWH), a tertiary gynaecological unit, where leiomyoma was documented as a primary indication for 43% of all hysterectomies carried out for benign gynecological causes during the study period (January–December 2016).

### 2.1. Data Collection

Collected patient data included the following: age, ethnicity, menopausal status, body mass index (BMI), parity, presenting symptoms, finding reported on preoperative imaging (ultra-sonographic scans (USS), CT, MRI), such as size of mass (if multiple masses present, the greatest diameter of largest mass was obtained), preoperative endometrial biopsy histology and preoperative blood results, including full blood count, LDH, and CA-125. Imaging and endometrial biopsies were obtained preoperatively, in the 3 months prior to hysterectomy. Blood tests were obtained 24 h prior to hysterectomy. Data were collected from electronic patient record databases and paper-based medical records and were anonymised prior to analysis. The LWH clinical team collected all the data included in this study, as part of service evaluation, assessing the standard of care at LWH, and none of the patient identifiers were included. This study was approved by the gynaecological directorate audit committee at LWH (Service Evaluation Proposal submitted/approved by LWH Audit committee, approved on 14 September 2017) and did not require ethical approval from the adult research ethics committee.

### 2.2. Statistical Analyses

Data were analysed using SPSS 22.0 (IBM SPSS for Windows. Armonk, NY, USA). To investigate the relationship between the preoperative variables and the histological diagnosis of LMS, we performed a Chi-squared test or Fisher’s Exact test and estimated the odds ratio (OR) and 95% confidence interval (CI) on the statistically significant results. A *p* value of <0.05 was considered to be statistically significant. Multivariate analysis was not carried out because of the small number of outcomes in the dataset (*n* = 31 LMS). Subgroup analysis based on menopausal status was not performed due to the small sample size.

## 3. Results

From a total of 32 preoperative variables (Supplementary Table S1), 7 were found to be significantly associated with increased odds of LMS (see Table 1). Patient demographics can be seen in Table 2.

### 3.1. Menopausal Status and Symptomatalogy

LMS group had a higher proportion of postmenopausal women (*p* < 0.001). Presenting symptoms such as pressure and postmenopausal bleeding (PMB) were more frequently associated with the LMS group (*p* = 0.002, OR 2.7, CI 1.61–5.05, and *p* = 0.001, OR 5.01, CI 1.62–15.54, respectively, Table 1).

### 3.2. Imaging

Considering the size of the uterine mass on imaging, a diameter greater than 10 cm was significantly associated with LMS (*p* < 0.0001, OR 8.52, CI 3.13–23.2). Women with LMS were significantly more likely to have had a CT/MRI scan performed preoperatively than women with leiomyoma (*p* < 0.0001, OR 9.89, CI 4.06–24.11), which emphasizes the importance of a clinician’s suspicion in investigation of LMS. In cases of LMS with MRI (*n* = 9), the reports commonly included the following features: haemorrhage (33%), necrosis (22%), and heterogeneous enhancing signal (55%). A standardized report proforma was not used. All MRI reports included the size and number of masses; however, only 5/9 included signal characteristics, 2/9 reported on necrosis or degeneration, and no reports commented on the perfusion pattern.

### 3.3. Endometrial Biopsy Result

A preoperative endometrial biopsy with a histological report of nuclear atypia or neoplasia was more likely in the LMS group (*p* = 0.001, OR 9.6, CI 2.02–45.4). In two of the cases with endometrial biopsy results suggestive of neoplasia, the reports stated “malignant neoplasm, widespread necrosis, sheets and islands of round and spindle cells,” and “poorly differentiated malignant epithelioid tumour of unknown origin.” In two other cases, LMS was diagnosed on preoperative endometrial biopsy; therefore, these were not included in the endometrial biopsy analysis.

### 3.4. Blood Test Results

**Figure 1:**
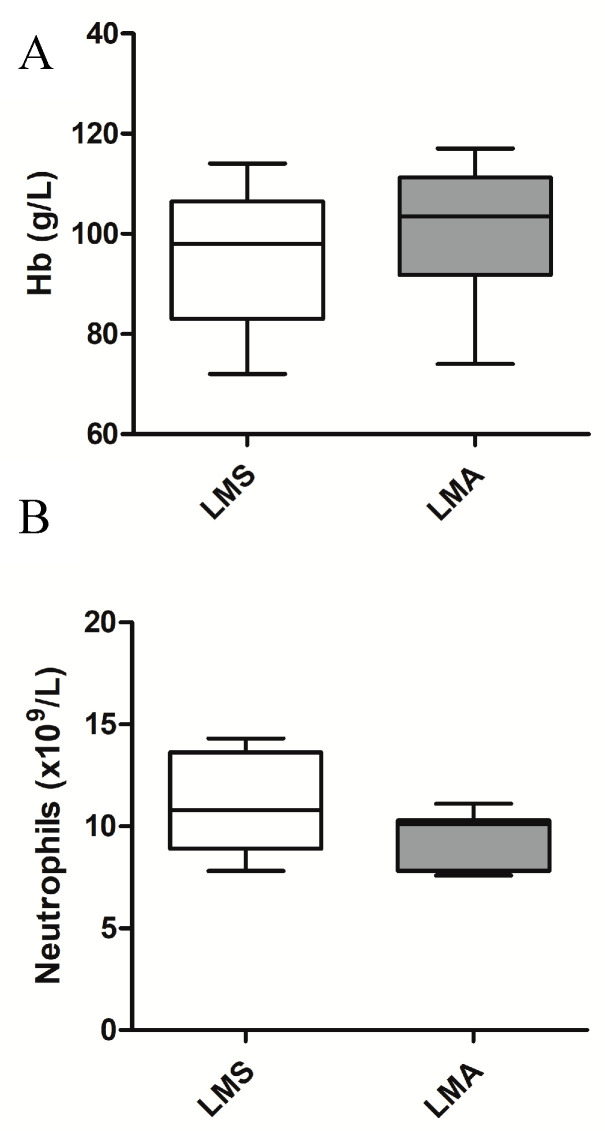
Box and whisker plots representing low haemoglobin (Hb) and neutrophilia between leiomyoma (LMA) and leiomyosarcoma (LMS) groups. (**A**) Hb results for LMS and LMA groups. LMS: Median = 98, 25% = 83, 75% = 106.5, Min = 72, Max = 114, (*n* = 17). LMA: Median = 103.5, 25% = 91.8, 75% = 111.3, Min = 74, Max = 117, (*n* = 28); (**B**) Neutrophil counts for LMS and LMA groups. LMS: Median = 10.8, 25% = 8.9, 75% = 13.6, Min = 7.8, Max = 14.3, (*n* = 10). LMA: Median = 10.1, 25% = 7.8, 75% = 10.3, Min = 7.6, Max = 11.1, (*n* = 9). Hb—haemoglobin, LMS—leiomyosarcoma, LMA—leiomyoma.

Abnormalities in preoperative full blood count results were significantly associated with LMS, including a low haemoglobin (Hb) level, defined as Hb < 118 g/L (*p* = 0.037, OR 2.22 CI 1.04–4.74) and a neutrophilia, defined as neutrophils >7.5 × 10^9^/L (*p* < 0.001, OR 5.72, CI 2.06–15.87) (Figure 1). Normal reference ranges are detailed in Supplementary Table S2. CA-125 was performed in only 36% of women (*n* = 68) and did not show a significant difference between groups. LDH levels were not recorded for any of the women included in this study.

## 4. Discussion

**Figure 2:**
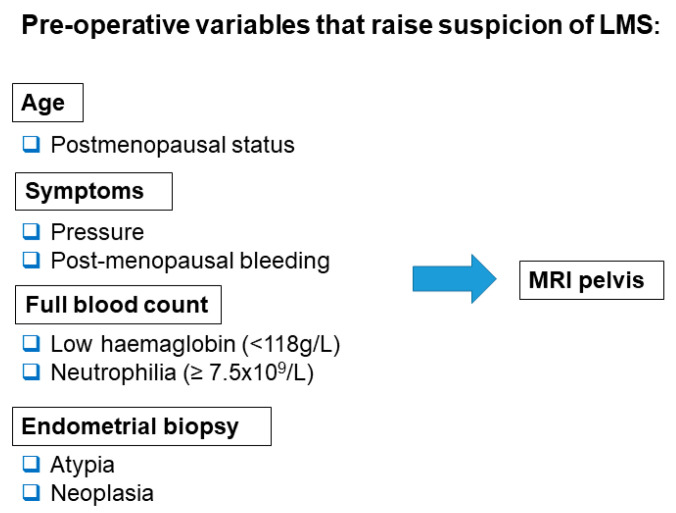
Pre-operative variables that should raise suspicion of LMS and prompt further imaging with MRI pelvis.

This study demonstrates that when women present with common symptoms suggestive of either leiomyoma or LMS, those at a higher risk of LMS can be identified preoperatively, using demographic and clinical features and routine, noninvasive investigations. We propose that further investigation with MRI is warranted to exclude LMS when the 7 preoperative parameters we have identified are present in women with presumed symptomatic leiomyomas (Figure 2).

In keeping with current published literature, leiomyomas were more common in premenopausal women than in postmenopausal women in our study, while the opposite trend was observed in LMS [3,5]. Presenting symptoms such as PMB and pelvic pressure were significantly associated with LMS; hence, we propose that simultaneous presentation of PMB and pressure indicate the need for further investigation, with LMS as a differential diagnosis. Although we did not find age to be a significant differential feature for diagnosis of LMS in our study as others have [25], we did find that postmenopausal status was significantly associated with LMS. Increasing age may not have been of significance in this study as the population was relatively young. When older women, particularly those above 60 years of age, who present a decade or more after menopause, with an enlarged uterus, malignancy is routinely considered, as symptomatic leiomyomas are not frequently seen in this age group. However, an enlarged uterus with suspected leiomyoma is a regular feature in perimenopausal women, and thus often considered to be an incidental finding. The women included in our study were relatively young and at the end of their reproductive life, which we believe to be the clinically most useful time to discern benign leiomyomas from LMS. Therefore, our data identify determining variables in this particular age group, which is clinically relevant.

Blood tests are often performed preoperatively in suspected leiomyoma/LMS [26], but their value in differentiating between the two diagnoses has not yet been fully elucidated. Our data demonstrated that low Hb and neutrophilia are significantly associated with LMS. Our finding of an increased neutrophil count being associated with LMS is in keeping with published studies, which report a raised neutrophil-to-lymphocyte ratio and neutrophilia in preoperative diagnosis of LMS [27,28]. Further, preoperative findings of an elevated neutrophil-to-lymphocyte ratio has been associated with poor prognosis in other soft tissue sarcomas, and significantly associated with tumour size ≥5 cm [29]. A clear explanation of the role of neutrophilia in soft tissue sarcomas such as LMS has not yet been elucidated; however, some have proposed theories including the ability of neutrophils to produce proangiogenic factors [30,31], allowing tumour growth and angiogenesis. Further work is needed to clarify the relationship between neutrophilia and LMS. There are limited published data on the predictive role of CA-125 in LMS. Some studies report a potential role in differential diagnosis between early-stage and advanced-stage LMS and survival [32,33] and other larger studies reporting no predictive value for CA-125 in LMS and no association between stages [21]. We found no significant association between CA-125 and preoperative diagnosis of LMS in this study. As a CA-125 was only performed in a small number of our study population, we cannot reliably comment on the use of this as a preoperative tool for LMS. Although previous researchers have proposed LDH levels to be a beneficial biomarker with a readily accessible and low-cost test [34,35], no women in our cohort had an LDH recorded preoperatively, and therefore we were unable to assess its diagnostic validity for LMS.

Given that uterine LMS arises from the deep muscular myometrial layer of the uterus rather than the superficial, easily accessible endometrium, an endometrial biopsy is thought to be of limited clinical value. Although there are studies reporting low sensitivity in detecting LMS in endometrial biopsy [1,35,36], our data suggest that our finding of atypical cells or neoplasia (without a known origin) on endometrial biopsy is significantly associated with LMS and likely to have some clinical value in preoperative diagnosis. Our findings are consistent with a large retrospective study reported preoperative endometrial sampling that suggested an invasive tumour in 86%, and correctly predicted the histological diagnosis in 64% [23].

There are no currently validated radiographic criteria to differentiate between a leiomyoma and LMS [24,37]. MRI is considered to have moderate diagnostic accuracy in LMS [8,38] and can identify discriminating features of LMS such as degeneration or haemorrhage within the uterine mass [39]. Although some studies report that diffusion-weighted MRI can differentiate between the two diseases with high sensitivity [40], it is not universally available, and furthermore, more recent robust studies have identified that contrast-enhanced MRI has a higher diagnostic accuracy in reference to leiomyomas and LMS [41]. Agreeing with multiple other studies, our data illustrated that a greatest diameter of the largest uterine mass of >10 cm was associated with LMS [10,24,42]. A mass of <10 cm was more likely to be a leiomyoma. There is conflicting evidence on the efficacy of rate of tumour growth in differentiating between leiomyoma and LMS [24,34], and this was not analysed in our study; therefore, we cannot comment on its value in preoperative diagnosis of LMS. In this study, only 9 women with LMS had a preoperative MRI performed. Analysis of specialist radiological features of LMS on MRI imaging has been studied previously [43], and of the MRI reports available for patients included in our study, features such as necrosis, haemorrhage and heterogeneous signal intensity were often present. However, not all of the features associated with LMS were commented upon, and a standardized reporting proforma was not used.

LMS is a rare disease, and published studies thus tend to contain a small number of cases usually without a suitable control group. We present consecutive, contemporaneous data from a single, large specialist gynecological unit and included a relatively large cohort of women with LMS, as well as a comparator group of women with symptomatic benign leiomyomas, with a comprehensive amount of associated preoperative clinical variables. However, some of our confidence intervals are wide due to small numbers; therefore, some results may lack precision. It should be noted that the control group was comprised of all women with symptomatic leiomyoma undergoing hysterectomy; therefore, there is a need for future studies to prospectively evaluate these findings in women with suspected leiomyomas undergoing other treatment options. The clinical relevance of our findings should be validated in a prospective larger study of women with suspected leiomyoma, in a combined risk stratification model, where multivariate analysis can be utilised to create a clinical checklist that could stratify women for their risk of having LMS.

## 5. Conclusions

Our study has identified a comprehensive panel of preoperative clinical features, biomarkers, diagnostic tools that differentiate between benign leiomyoma and LMS, which could easily be incorporated into clinical practice for risk stratification of women with occult LMS.

## Figures and Tables

**Table 1:** Comparison of preoperative parameters between leiomyoma and LMS groups.

Parameter	No. LMA (%) *n* = 159	No. LMS (%) *n* = 31	*p*-Value	OR (95% CI)
Postmenopausal	39 (24.7)	30 (56.7)	<0.001	3.08 (1.61–5.91)
Pressure	24 (15.1)	31 (38.7)	0.002	2.7 (1.45–5.05)
PMB *	13 (33.3)	17 (82.4)	0.001	5.01 (1.62–15.54)
Atypia/neoplasia	5 (6.4)	10 (40)	0.001	9.6 (2.02–45.5)
Hb < 118 g/L	28 (25)	30 (56.7)	0.037	2.22 (1.04–4.74)
Neutrophil count ≥ 7.5 × 10^9^/L	9 (8)	30 (33.3)	<0.001	5.72 (2.06–15.87)
Mass ≥ 10 cm	30 (29.1)	27 (77.8)	<0.001	8.52 (3.13–23.2)

LMA = Leiomyoma. LMS = Leiomyosarcoma. OR = Odds radio. 95% CI = Confidence interval. PMB = Postmenopausal bleeding. Hb = Haemoglobin. * = Sample size excluded premenopausal women.

**Table 2:** Patient demographic variables between leiomyoma and LMA groups.

Demographic	Subcategory	No. LMA (%)	No. LMS (%)	*p*-Value
Age	31–40 years	11 (6.9)	2 (6.5)	0.28
	41–49 years	90 (56.6)	13 (46.9)	
	>50 years	58 (36.5)	16 (51.6)	
Ethnicity	White British	141 (88.7)	26 (96.3)	0.53
	Asian British	6 (3.8)	1 (3.7)	
	Black British	2 (1.3)	0	
	Other	10 (6.3)	0	
Body Mass Index (kg/m^2^)	<20	4 (2.5)	0	0.62
	20–29	84 (52.8)	17 (56.7)	
	30–39	62 (40)	10 (33.3)	
	>40	9 (5.7)	3 (10)	
Parity	Nulliparous	10 (9.1)	5 (20)	0.44
	1	23 (20.9)	2 (8)	
	2	49 (44.6)	13 (52)	
	3	21 (19.1)	4 (16)	
	4	5 (4.6)	1 (4)	
	5+	2 (1.8)	0	
Menopausal status	Premenopausal	119 (75.3)	13 (34.3)	<0.001
	Postmenopausal	39 (24.7)	17 (56.7)	

LMA = Leiomyoma. LMS = Leiomyosarcoma.

## Supplementary Materials

Supplementary materials can be found at https://www.mdpi.com/2075-4418/10/10/735/s1. Supplementary Table S1: Summary of all pre-operative variables in leiomyoma and LMS groups. Supplementary Table S2: Normal reference ranges for blood results.
