# Supplementary material for: Developing a Preoperative Algorithm for the Diagnosis of Uterine Leiomyosarcoma
Source: Diagnostics (Basel). 2020 Sep 23;10(10):735. doi: 10.3390/diagnostics10100735 (PMC7598216; doi:10.3390/diagnostics10100735)
Supplement: Supplementary file 1 [file diagnostics-10-00735-s001.pdf]

diagnostics

Supplementary Table S1: Summary of all pre-operative variables in leiomyoma and LMS groups.

Category	Pre-operative variable	No. LMA	LMA sample size	Frequency in LMA (%)	No. LMS	LMS sample size	Frequency in LMS (%)	P-value
**Menopausal status**	**Postmenopausal**	39	158	24.7	17	30	56.7	<0.001
**Symptoms and indications**	**Menorrhagia***	94	119	79	11	13	84.6	0.633
	**PMB***	13	39	33.3	14	17	82.4	0.001
	**Pelvic pain**	40	159	25.2	12	31	38.7	0.122
	**Pressure**	24	159	15.1	12	31	38.7	0.002
	**Urinary symptoms**	18	159	11.3	4	31	12.9	0.801
	**Suspected fibroids**	73	159	45.9	8	31	25.8	0.038
	**Prolapse**	14	159	8.8	0	31	0	0.086
**Previous surgery**	**Abdominal – laparoscopy**	48	159	30.2	5	27	18.5	0.214
	**Abdominal – laparotomy**	43	159	27	8	27	29.6	0.781
	**Vaginal – prolapse repair**	2	159	1.3	1	27	3.7	0.351
**Comorbidities**	**Cardiovascular disease**	41	159	25.8	7	29	24.1	0.852
	**Respiratory disease**	24	159	15.1	3	29	10.3	0.502
	**CNS disease**	11	159	6.9	3	29	10.3	0.518
	**GI disease**	35	159	22	7	29	24.1	0.800
	**Chronic urinary problems**	11	159	6.9	2	29	6.9	0.997
	**Previous malignancy**	8	159	5	1	29	3.5	0.713
	**Known endometriosis**	22	157	14	1	30	3.3	0.103
**Previous treatment for fibroids**	**Ineffective**	93	106	87.7	13	13	100	0.181
**Endometrial biopsy result**	**Proliferative**	29	78	37.2	1	10	10	0.088
	**Secretory**	25	78	32.1	1	10	10	0.150
	**Hyperplasia**	3	78	3.9	0	10	0	0.528
	**Atypical cells**	5	78	6.4	2	10	20	0.139
	**Neoplasia**	0	78	0	2	10	20	0.012
	**Atypia/neoplasia combined**	5	78	0	4	10	40	0.001
**Preoperative bloods**	**Low hemaglobin (<118g/L)**	28	112	25	17	30	56.7	0.037
	**Low haematocrit (<0.36L/L)**	37	112	33	17	30	56.7	0.283
	**High neutrophil count (>7.5x10^9/L)**	9	112	8	10	30	33.3	<0.001
	**Abnormal CA-125**	15	58	25.9	4	10	40	0.357
	**Abnormal LDH**	0	0	n/a	0	0	n/a	n/a
	**Abnormal lymphocyte count**	7	99	7.1	2	25	8	0.873
**Radiological imaging**	**Size of mass ≥ 10cm**	30	103	29.1	21	27	77.8	<0.0001
	**Multiple masses**	67	106	63.2	6	8	75	0.503

Key: LMA = Leiomyoma. LMS = Leiomyosarcoma. PMB = Post-menopausal bleeding. Hb = Hemoglobin. LDH = Lactate dehydrogenase. *= Sample size excluded pre-menopausal women.

Supplementary Table S2: Normal reference ranges for blood results.

Parameter	Normal reference range
Haemaglobin	118-148 g/L
Haematocrit	0.36-0.44 L/L
Neutrophil count	2-7.5 x 10^9/L
